# M. Valleix

**Published:** 1855-12

**Authors:** 


### *M. Valleix.*

The recent death of the distinguished Dr. Valleix, who died on the 12th of July last, in the midst of a career of usefulness and success, is an event which has caused a deep sensation in Paris, where he was not only appreciated as a man of eminent talents, but esteemed by all who knew him for his amiable character and agreeable manners. Born in Toulouse in 1807, he commenced the study of medicine in Paris, in 1830, and received his diploma in 1835. He formed an early attachment to M. Louis, and became, during his whole life, a most ardent follower of the "numerical system," his attachment to which was only equalled by that which he bore to his revered master.

While *interne* of the hospitals, Valleix became an inmate of the Foundling Hospital, where his attention was first directed towards the study of the diseases of infancy. The results of his observations in this department, besides a very large number of detached papers published in various journals, consist chiefly of the *Clinique des maladies des Enfants nouveau-nes*, an octavo volume of 700 pages; and the *Recherches sur la Frequence du pouls chez les Enfants nouveau-nes, &c.*, in the Memories of the Society of Observation. In 1838, M. Valleix began to investigate the difficult subject of neuralgia, and in 1841 published his well-known work, the *Traite des Nevralgies, ou Affections Douloureuses des Nerfs*, an octavo of 720 pages, which received a prize of 3000 francs from the Academy of Medicine. In 1844 his essay on *Œdema of the Glottis* received a prize of 1500 francs, from the same learned body. Shortly after appeared the first volue of the *Guide du Medecin Praticien*, a work in ten volumes, which was not completed until 1848. The success of this work is shown by the fact that a second edition was published in 1851, and a third in 1853. In 1851 he promulgated in his clinical lectures at La Pitie, the results of his observations on certain diseases of the uterus, especially its displacement, and their treatment by means of a modification of Simpson's intra-uterine sound. His views met with much opposition, and became the subject of one of the most interesting discussions which ever took place in the *Academic de Medecine*. M. Valleix was occupied, shortly before his death, in collecting new observations on this subject, in view of deciding several questions which arose in the course of the debate. He was actually engaged at the time of his fatal illness in preparing a paper on the co-existence of bronchitis with emphysema and diseases of the heart; and also another on the results obtained by percussion in pleurisy. His various contributions to the journals, including original memoirs, critical reviews, analyses of books and controversial articles, would fill several large volumes.

The editor of the *Archives Generales de Medecine*, in a notice of M. Valleix, from which we have derived the above facts, thus concludes: "M. Valleix was in truth one of the most intelligent disciples of M. Louis. He made the best use of the doctrines of this celebrated master, when the latter, overwhelmed by practice, could no longer sustain them by his own investigations. By his labors and his criticisms he showed how ill founded was the opposition made to them. Without falling into those exaggerations which may compromise the best cause, he boldly supported the principles of that philosophy on which alone the progress of medicine depends, and clearly distinguished what are often confounded, the slow and painful growth of medical science, and its practical requirements, so frequently destitute of scientific aid. In a word, M. Valleix was the successor of M. Louis, a relation which constituted his principal task, and which will be his chief glory."—*Boston Med. & Surg. Journal.*

